# Ultrastructural characterization of peri-synaptic astrocytic processes around cerebellar Purkinje spines under resting and stimulated conditions

**DOI:** 10.1186/s13041-025-01198-7

**Published:** 2025-03-31

**Authors:** Jung-Hwa Tao-Cheng

**Affiliations:** NINDS Electron Microscopy Facility National Institute of Neurological Disorders and Stroke, National Institutes of Health, Bethesda, MD 20892 USA

**Keywords:** Tripartite synapses, Electron microscopy, Astrocyte, Purkinje spines, Glutamate uptake, Glutamate transporter, Synaptic cleft

## Abstract

In mammalian brains, astroglia presence near glutamatergic excitatory synapses has generated the term “tripartite” junctions, based on the close association of astrocytic processes near the active zone formed by presynaptic axonal terminal and postsynaptic dendritic spines. One major function of these astrocytic processes is to take up glutamate that spill out of the synaptic cleft during activity, via glutamate transporters located on astroglial plasma membrane. Comapred to other regions of the brain, the cerebellar Purkinje spines in the molecular layer are virtually completely ensheathed by Bergman glia, a special type of astrocyte, unique to cerebellum. The present electron microscopy study classifies these peri-synaptic astrocytic processes (PAP) ensheathing the Purkinje spine synapses into three types based on structural criteria: (1) Type 1– astrocytic process is situated at the edge of the synaptic cleft immediately next to the synaptic active zone. Under fast perfusion fixation conditions where synapses were under resting states, ~ 58% of the PAP’s were scored as Type 1. The occurrence frequency of Type 1 PAP significantly decreased to 25% upon a 5–8 min delay in perfusion fixation, where synapses were under stimulated states. (2) Type 2– astrocytic process covers part of the postsynaptic membrane containing the postsynaptic density (PSD), so that this part of the PSD is separated from its presynaptic terminal. Occurrence frequency of Type 2 PAP’s significantly increased from ~ 14% under fast perfusion fixation to 31% upon delayed perfusion fixation, and the average length of the PSD edge covered by astroglia increased from 41 nm to 57 nm upon delayed perfusion fixation. (3) Type 3– astrocytic process is situated some distance away from the active zone, while the presynaptic axon terminal extends to enwrap the spine beyond the active zone. Occurrence frequency of Type 3 PAP’s increased from 28 to 43% upon delayed perfusion fixation, and the average length between apposed axon terminal and spine beyond the synaptic cleft significantly increased from 98 to 209 nm upon delayed perfusion fixation. Thus, upon stimulation, the tripartite synaptic junctions undergo dynamic structural changes with the astrocytic processes moving into the open cleft to cover the exposed postsynaptic membrane containing PSD, the presynaptic axon terminals extending to wrap the postsynaptic spine beyond the synaptic cleft. Both structural changes may facilitate glutamate uptake to clear the transmitter spilled out from the synaptic cleft during intense activity and prevent damage from overstimulation.

**Supplementary Information:**

The online version contains supplementary material available at 10.1186/s13041-025-01198-7.

## Introduction

Astroglia presence near glutamatergic excitatory synapses in mammalian brain is involved in trophic support and modulation of synaptic transmissions [1–3]. These peri-synaptic astroglial processes (PAP) together with the nearby synaptic junctions formed between presynaptic axon terminal and postsynaptic spine have been termed as “tripartite synapses” [4], which display dynamic structural plasticity [1]. One major role of the close ensheathment of synapses by astrocytic processes is glutamate uptake [5], achieved through abundant glutamate transporters (EAAT, excitatory amino acid transporters) on astroglial plasma membranes [5–7].

Notably, the degree of ensheathment of synapses by astroglia varies among different regions of the brain. Cerebellar Purkinje spine is known to be completely ensheathed by Bergman glia [8, 9], a particular type of astroglia in molecular layer of the cerebellum [10]. This complete ensheathment has been shown to regulate synapse numbers [11], and knocking out astroglial glutamate transporters affects the development and maintenance of cerebellar Purkinje spine synapses [12]. In contrast, astroglial ensheathment of the spine is only partial in other parts of the brain, e.g., in cerebral cortex [9, 13], and in hippocampus [1, 14]. Furthermore, the degree of astroglial ensheathment of synapses is affected by stimulation or diseases. For example, the degree of coverage of the synapses by PAP in cerebral cortex corelates with synaptic strength [15], and increases after ischemia-induced spreading depolarization [16]. In hippocampus, the degree of astroglial coverage of synapses increases upon epileptogenesis [17], and the tip of the PAP retracts from the synaptic cleft upon fear memory conditioning [14], but not in a mouse model of Alzheimer’s disease [18]. Additionally, decreased glial coverage of spines in striatum was documented in a mouse model of Huntington’s disease [19]. The functional implications of the changes in astroglial association with synapses are centered on glutamate uptake near synapses to either increase or decrease the clearing of transmitter from the synaptic cleft.

Notably, the great majority of the above mentioned studies on activity- or disease-induced changes on degrees of astroglial ensheathment of the synapses required meticulous and labor-intensive 3D reconstruction from serial EM analysis. The present study took advantage of the fact that cerebellar Purkinje spine heads are completely ensheathed by its presynaptic terminal and Bergman glia in each and every thin sections whether under resting or stimulated conditions. Therefore, every such synapse is a *bona fide* tripartite synapse, and any changes in the ensheathment arrangement such as the retraction or extension of the axon and glia around the spine can be credibly documented by examining a large number of synaptic profiles in single sections. A reasonable assessment on these structural changes can be achieved from 2D images without the necessity of 3D reconstruction of serial images [20].

Here, classic transmission EM images were examined at high magnification for the structural organization among the three partners of the tripartite synapse under resting vs. stimulated conditions. The PAP’s were classified into three types based on their relative location, configuration, and distance to the synaptic cleft. The occurrence frequency of the three types of PAP’s were scored and found to be significantly altered upon stimulation, and the functional implications of these stimulation-induced structural changes were discussed.

## Materials and methods

### Perfusion fixation of mouse brains

Samples from six perfusion-fixed mouse brains from a previously published report [21] were reexamined here for structural changes of astroglial ensheathment of cerebellar Purkinje spine synapses. Briefly, adult male mice, 25–35 g in weight, were deeply anesthetized with isoflurane and perfusion fixed through the heart with 2% glutaraldehyde + 2% paraformaldehyde in 0.1 M sodium cacodylate buffer at pH7.4 for four C57 black mice, or first with 3.75% acrolein + 2% paraformaldehyde in PBS, then followed by 2% paraformaldehyde in PBS for two NIH Swiss mice. The time interval starting from the moment the diaphragm was cut to the moment when the outflow from the atrium turned from blood to clear fixative was recorded. Those animals that were successfully perfused within 100 s were classified as “fast” perfusion. For the “delayed” perfusion experiments, calcium- and magnesium-containing PBS was first perfused through the heart for 5–8 min before the start of the fixative. Neurons were mostly under resting state after fast perfusion, while delayed perfusion fixation promoted an ischemic excitatory state [21]. The perfusion-fixed cerebellums were dissected and vibratomed into 100 μm thick coronal slices and stored in 2% glutaraldehyde in 0.1 M cacodylate buffer at 4˚C.

### Electron microscopy

Samples were post-fixed with 1% osmium tetroxide in 0.1 M cacodylate buffer for 1 h on ice and *en bloc* stained with 1% uranyl acetate in 0.1 N acetate buffer at pH 5.0 overnight at 4˚C. Samples were then dehydrated in a graded series of ethanol and embedded in epoxy resins. Thin sections were cut at ~ 70 nm and counterstained with lead citrate. Images were photographed on a JEOL 1200 EX transmission electron microscope at 60 KV with a bottom-mounted digital CCD camera (AMT XR-100, Danvers, MA, USA).

### Morphometry

Glutamatergic, asymmetric synapses were identified by their characteristic presynaptic clusters of synaptic vesicles, postsynaptic density (PSD) and the synaptic cleft (a rigid ~ 20 nm apposition between the pre- and post-synaptic membranes at the active zone) [22]. Sampling of synapses was restricted to the molecular layer of the cerebellum, where every asymmetric synaptic profile with a cross-sectioned PSD was photographed from randomly selected grid openings until at least ~ 30 synaptic profiles were collected at a magnification of 40,000X. In this region of interest, all asymmetric synapses are formed by parallel fibers from granular cells with Purkinje spines, and the spine heads were completely ensheathed by its presynaptic terminal and Bergman glia [8, 9]. For comparison, asymmetric synapses from stratum radiatum of the hippocampal CA1 region were also examined.

**Fig. 1:**
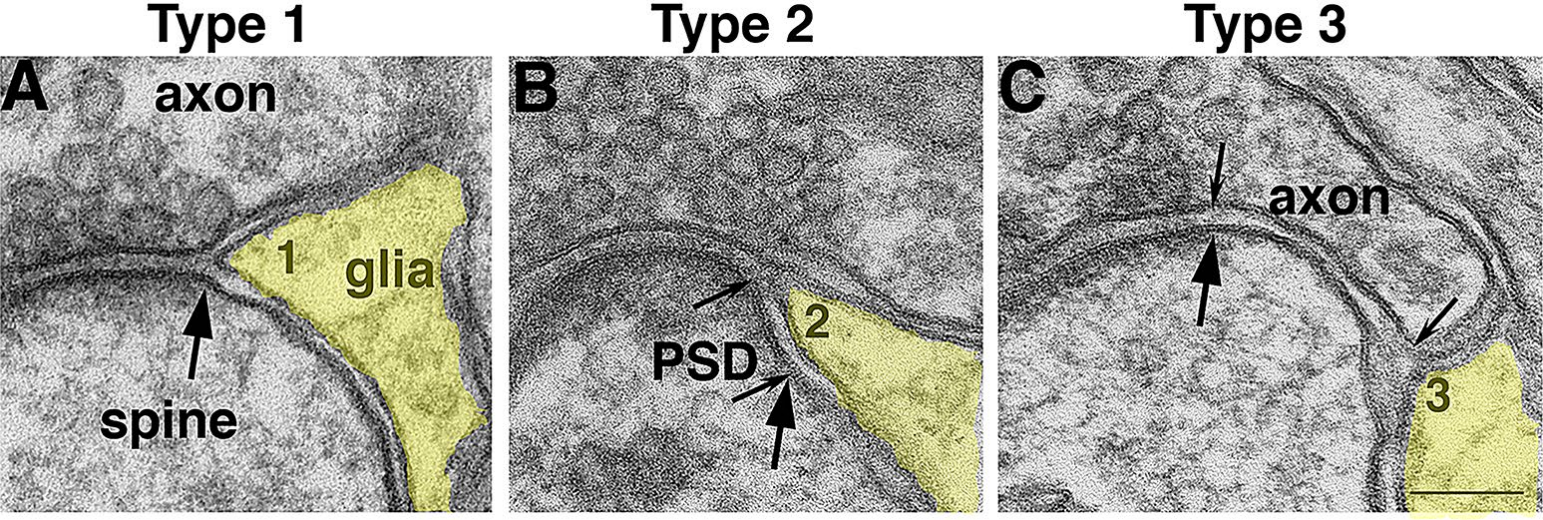
Classification of three types of peri-synaptic astrocytic processes (PAP, shaded in yellow) at Purkinje spines in cerebellar molecular layer. Large arrows point to the edge of the postsynaptic density (PSD), which is typically apposed by the presynaptic active zone with a uniform-distanced synaptic cleft containing filamentous materials. In Type 1 PAP (**A**), the astroglial process (marked as “1”) is at the edge of the synaptic cleft. The extracellular space between the glial and the axonal or spine membranes appears clear, and is typically narrower than the synaptic cleft. In Type 2 PAP (**B**), the astrocytic process (marked as “2”) covers a part of the PSD-containing postsynaptic membrane, with the area covered by glia marked by two small arrows. In Type 3 PAP (**C**), the astrocytic process (marked as “3”) is some distance away from the synaptic cleft, and the presynaptic terminal extends over the spine beyond the active zone. Two small arrows mark this extended area between axon and spine. Scale bar = 100 nm

The present study focuses on the peri-synaptic axon and astroglial ensheathment of Purkinje spines, and sampling is restricted to profiles where the three elements of the tripartite synapse are clearly discernible with cross-sectioned membranes, from which distances between elements can be measured. Each cross-sectioned synaptic profile contained one synaptic cleft with two edges, and each edge of the synaptic cleft was scored and classified by structural characteristics into three types (Fig. 1): (1) Type 1– astrocytic process is situated at the edge of the synaptic cleft immediately next to the active zone (Fig. 1A), with zero distance between the tip of the glia and the edge of the synaptic cleft. (2) Type 2– astrocytic process covers part of the postsynaptic membrane containing the PSD (Fig. 1B) at the edge of the synaptic cleft. This type of configuration is termed as “open cleft” [23] where the edge of the postsynaptic membrane containing the PSD was separated from its presynaptic terminal. The distance of the PSD covered by glia was measured as “length of contact area of Type 2” (marked between two small arrows in Fig. 1B). Length was measured on marked micrographs with ImageJ (National Institutes of Health, Bethesda, MD, USA). (3) Type 3– astrocytic process is situated some distance away from the synaptic cleft (Fig. 1C), with the presynaptic terminal extending beyond the active zone. The distance of the extended axolemma is measured as “length of contact area of Type 3” (marked between two small arrows in Fig. 1C). Every edge of the synaptic cleft falls into one of the three types, and there is no overlapping among the three types.

### Statistical analysis

Within each pair of animals, statistical significance on frequency of each type of PAP was evaluated by Chi-Square test of independence between the fast and the delayed perfusion-fixed samples. For length of contact areas, mean values were evaluated by Student’s t-test between the two groups, and median values were evaluated by Wilcoxon’s test.

## Results

### Ultrastructural characterization of peri-synaptic astrocytic processes (PAP) at cerebellar purkinje spines under resting conditions

**Fig. 2:**
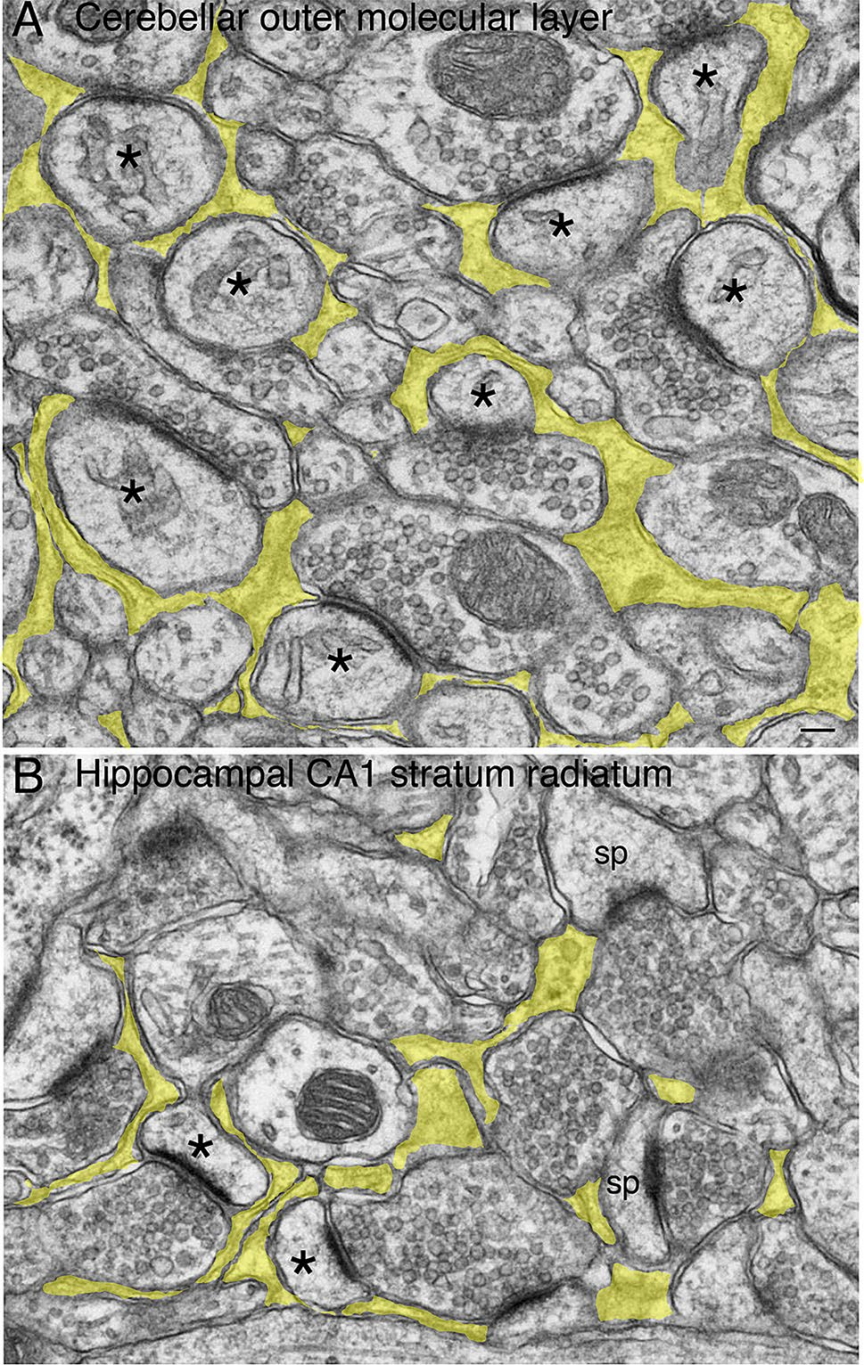
Astrocytic processes (shaded in yellow) in cerebellar molecular layer (**A**) and hippocampal CA1 stratum radiatum (**B**). (**A**) Virtually all Purkinje spines (asterisks) were completely ensheathed by Bergman glia, while spines in (**B**) can range from completely (asterisks) to partially (sp) ensheathed by astrocytic processes. Scale bar = 100 nm

The great majority of synapses in cerebellar molecular layer consists of parallel fibers from cerebellar grannular cells synapsing with Purkinje spines, which are completely ensheathed by Bergman glia, a special type of astrocyte, unique to cerebellum [8] (Fig. 2A). In contrast, in stratum radiatum of hippocampal CA1 region, astrocytic processes were often seen at peri-synpatic locations but not ensheathing the entire perimeter of the spine head [1, 14] (Fig. 2B). The hippocampal spines can be juxtaposed with neuronal elements like axons or dendrites, or oligodendrocytes (Supplementary Fig. 1). Notice also that Purkinje spines were typically roundish in a grape-like shape and always contained endoplasmic reticulum (ER) [24] (Fig. 2A), whereas the hippocampal spines were heterogenous in size and shape with much fewer ER [25] (Fig. 2B).

### Structural changes of PAP’s at cerebellar Purkinje spines upon stimulation

A 5–8 min delay in perfusion fixation caused the neuronal tissue to be under excitatory conditions [21], triggering increases in thickness and curvature of the PSD, and reorganization of ER arrangements. The present report focuses on the structural changes of PAP’s at Purkinje spines under stimulated conditions upon delayed perfusion fixation.

### Changes in occurrence frequencies of the three types of PAP’s

**Fig. 3:**
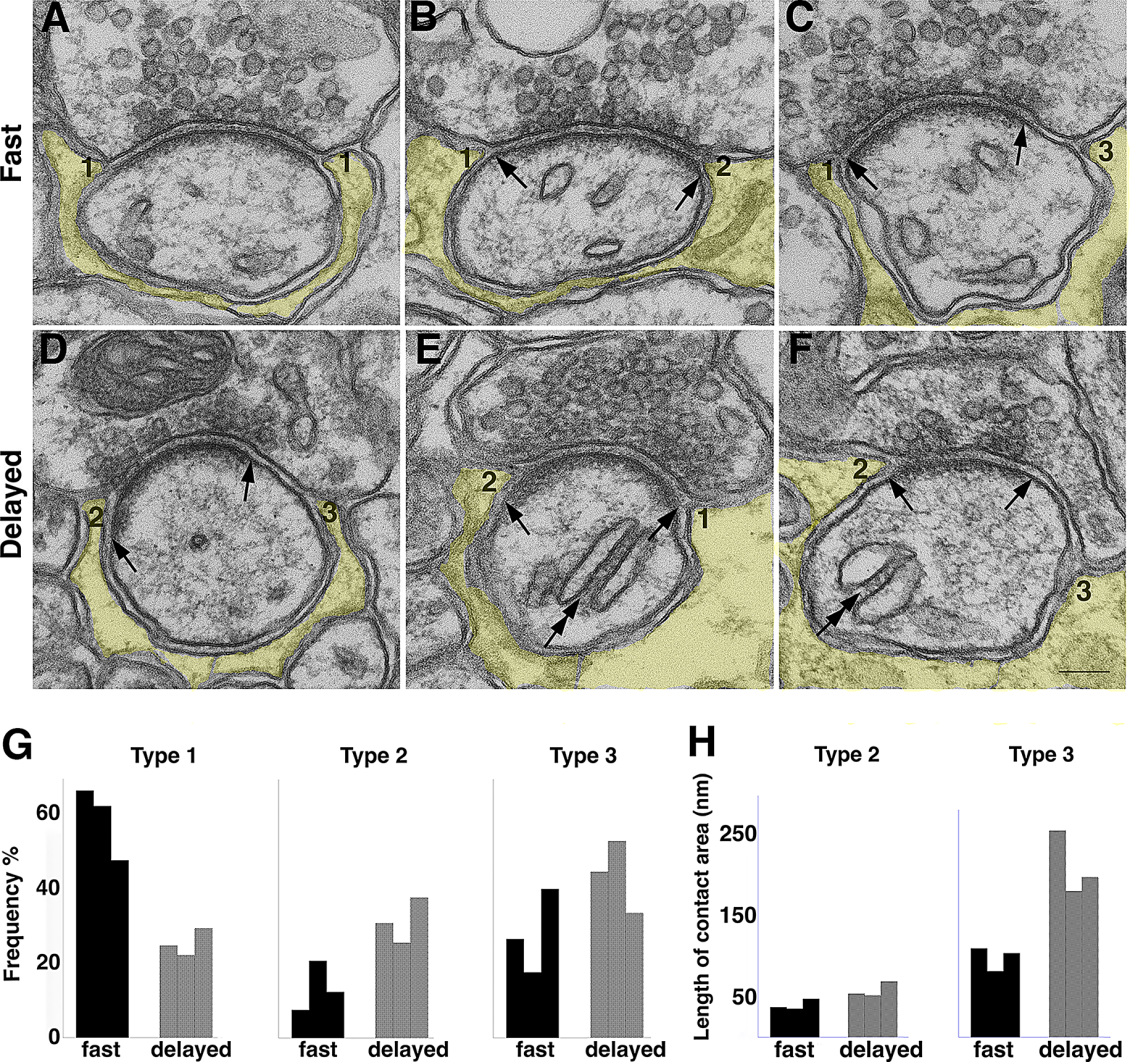
PAP’s (shaded yellow) ensheathing cerebellar Purkinje spines under resting (**A, B, C**; upon fast perfusion fixation) and stimulated (**D, E, F**; upon delayed perfusion fixation) conditions. Scale bar = 100 nm. Large arrows point to the edges of the PSD. Double arrows in E, F point to stacks of endoplasmic reticulum, an indication of delayed perfusion fixation [21, 26]. The three types of PAP’s are marked as 1, 2, 3 in A-F, and their occurrence frequencies are shown in bar graphs in G [from 3 animals each, under fast perfusion fixation (black bars) and delayed perfusion fixation (grey bars), data from Table 1]. (**H**) Upon delayed perfusion fixation, the length of contact area of Type 2 PAP (between glia and PSD containing postsynaptic membrane) did not change significantly, whereas the contact area of Type 3 PAP (between presynaptic terminal and spine beyond the synaptic cleft) significantly increased (Table 2)

The present study classifies the PAP’s ensheathing the Purkinje spines into three types based on structural criteria (cf. Fig. 1 in methods). Under fast perfusion fixation conditions where synapses were under resting states, (1) 58 ± 6% (mean of 3 experiments; Table 1) of the PAP’s were scored as Type 1, where the astroglial process was immediately adjacent to the edge of the synaptic cleft between the presynaptic active zone and the postsynaptic membrane containing the PSD (marked as “1” in Fig. 3A, B, C), (2) 14 ± 4% were scored as Type 2, where the astroglial process covered part of postsynaptic membrane containing the PSD (marked as “2” in Fig. 3B), and (3) 28 ± 7% were scored as Type 3, where the astroglial process was some distance from the edge of the synaptic cleft (marked as “3” in Fig. 3C). Notably, within a single cross-sectioned synaptic cleft, more than one type of PAP can co-exist as shown in Fig. 3B and C, indicating variable arrangements around the periphery of the active zone, suggesting a dynamic relationship among the three components of the tripartite junctions.

**Table 1:** Occurrence frequencies (%) of three different types of PAP at cerebellar purkinje spines under fast and delayed perfusion fixation conditions

		Fast Perfusion-fixation (*n*)	Delayed Perfusion-fixation (*n*)
**Type 1**	1st pair of animals	66% (53)	24.3% (70) *P* < 0.0001
	2nd pair of animals	61.9% (97)	22% (59) *P* < 0.0001
	3rd pair of animals	47.4% (57)	29.2% (72) *P* < 0.05
	**Mean ± SEM**	**58.4 ± 5.6**	**25.2 ± 2.1** *P* < 0.05
**Type 2**	1st pair of animals	7.5%	31.4% *P* < 0.005
	2nd pair of animals	20.6%	25.4% NS
	3rd pair of animals	12.3%	37.5% *P* < 0.005
	**Mean ± SEM**	**13.5 ± 3.8**	**31.4 ± 3.5** *P* < 0.05
**Type 3**	1st pair of animals	26.4%	44.3% *P* < 0.005
	2nd pair of animals	17.5%	52.5% *P* < 0.0001
	3rd pair of animals	40.4%	33.3% NS
	**Mean ± SEM**	**28.1 ± 6.7**	**43.3 ± 5.6** NS

See Methods (Fig. 1) for definition of the three types of PAP, and the scoring criteria

(n)– total number of synaptic cleft edges measured

Within each pair of animals, occurrence frequencies were compared by Chi-Square test of independence

Mean values were compared by Student’s t-test

NS– non-significant

**Table 2:** Mean and median values (nm) of length of contact area of type 2 and type 3 of PAP at cerebellar purkinje spines under fast and delayed perfusion fixation conditions

		Fast perfusion-fixation			Delayed perfusion-fixation		
		mean	median	(n)	mean	median	(n)
Type 2	1st pair of animals	37 ± 4	37	(4)	53 ± 4 *P* < 0.05	47 NS	(22)
	2nd pair of animals	35 ± 3	33	(20)	51 ± 8 NS	47 *P* < 0.05	(15)
	3rd pair of animals	49 ± 13	33	(7)	68 ± 6 NS	67 NS	(27)
	**Mean ± SEM**	**41 ± 5**			**57 ± 5** **NS**		
Type 3	1st pair of animals	109 ± 11	110	(14)	253 ± 30 *P* < 0.0001	227 *P* < 0.005	(31)
	2nd pair of animals	81 ± 10	80	(17)	179 ± 29 *P* < 0.005	113 *P* < 0.05	(31)
	3rd pair of animals	103 ± 11	87	(23)	196 ± 34 *P* < 0.05	140 NS	(24)
	**Mean ± SEM**	**98 ± 9**			**209 ± 23** *P* < 0.05		

See Methods (Fig. 1) for definition of different types of PAP, and the respective criteria for length measurements

(n)– number of synaptic cleft edges measured

Mean values were compared (fast vs. delayed) by Student’s t-test, median values were compared by Wilcoxon test

Upon delayed perfusion fixation where synapses were stimulated, occurrence frequencies of all three types of PAP changed when compared to the values under fast perfusion fixation conditions (serving as controls). The frequency of Type 1 PAP significantly decreased to 43% of controls, while frequency of Type 2 significantly increased to 233% of controls, and frequency of Type 3 increased to 153% of controls, though short of reaching statistical significance (Fig. 3G; Table 1). Notice that upon delayed perfusion fixation, ER cisternae in Purkinje spines often stacked up (double arrows in Fig. 3E, F), a benchmark structural change of neurons under excitatory conditions [26], especially prominent in cerebellar Purkinje cells [21]. Notably, regardless of whether samples were under resting or stimulated conditions, Purkinje spines were consistently only ensheathed by glia or its own presynaptic axon.

### Changes in length of contact area in type 2 and type 3 of PAP’s

For Type 2 PAP (cf. Figure 1B), the mean values of length of contact area between glia and the PSD-containing postsynaptic membrane was 41 ± 5 nm and 57 ± 5 nm under fast or delayed perfusion fixation conditions, respectively (mean of 3 animals, Table 2). Although this value increased by 39% upon delayed perfusion fixation (Fig. 3H left panel), it was short of reaching statistical significance. It has been reported that the average PSD length of the Purkinje spine synapses (at ~ 300 nm) did not change upon delayed perfusion fixation [21]. Here a ratio of glia-covered PSD length to the total PSD length was calculated, and there was no significant difference (10.6 ± 1.1% vs. 13.3 ± 1.6% under fast and delayed perfusion fixation, respectively, from 3 pairs of animals).

For Type 3 PAP (cf. Figure 1C), the mean value of length of contact area between presynaptic axon terminals and Purkinje spines beyond the edge of synaptic cleft was 98 ± 9 nm (mean of 3 animals, Table 2) under fast perfusion fixation conditions. Upon delayed perfusion fixation, this value significantly increased by 113% (Fig. 3H right panel) to 209 ± 23 nm.

**Fig. 4:**
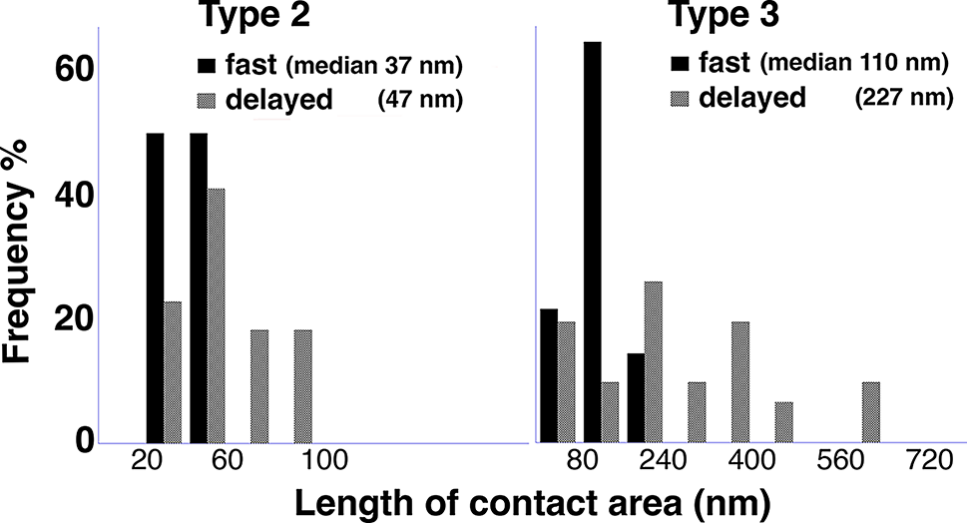
Histograms from one representative animal each under fast (black bars) and delayed (grey bars) perfusion fixation condition showing distribution patterns of the length of contact area for Type 2 (between glia and PSD-containing postsynaptic membrane) and Type 3 (between presynaptic terminal and postsynaptic spine beyond the synaptic cleft) PAP’s. Differences in median values was not significant for Type 2, and *P* < 0.005 for Type3 by Wilcoxon test (Table 2)

Histograms from one representative animal (the first pair of animals in Table 2) under each perfusion fixation condition are shown in Fig. 4. Because the distributions were skewed, a non-parametric test of the median values was used. Both types of contact area increased upon delayed perfusion fixation, but only Type 3 reached statistical significance.

One interesting feature of the Purkinje spines is that whenever there is a separation of the pre- and post-synaptic membranes forming an open cleft at the edge of the synaptic cleft [23], the exposed PSD-containing postsynaptic membrane was always apposed by an astrocytic process. This feature is not present at open clefts in hippocampus CA1 or cerebral cortex [23]. The length of such contact area of Type 2 PAP averaged ~ 40–60 nm (Table 2), and hardly exceeded 100 nm (Fig. 4, left panel).

On the other hand, the length of contact area of Type 3 PAP between presynaptic axon terminal and Purkinje spine beyond the edge of the synaptic cleft can be extensive to up to 650 nm upon delayed perfusion fixation (Fig. 4 right panel). This dramatic increase in contact area comes from the presynaptic terminal extending its process to wrap around the postsynaptic spine.

### Presynaptic axon terminals enwrap the Purkinje spines under stimulated conditions

**Fig. 5:**
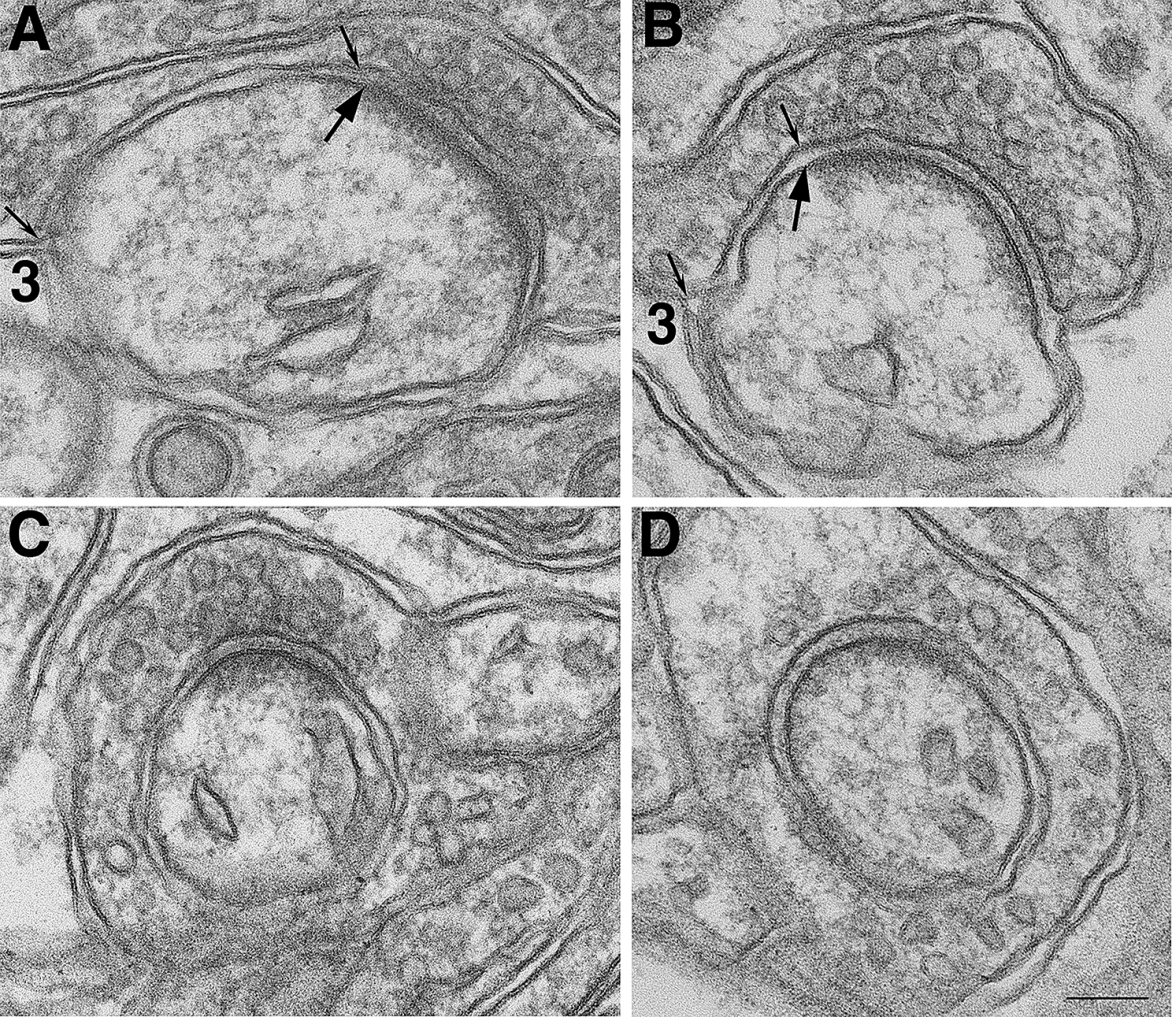
Examples of presynaptic axon terminals enwrapping spines in cerebellar molecular layer upon delayed perfusion fixation. Type 3 PAP (marked as “3” in **A, B**) was common in cerebellum, where presynaptic axon terminals enwrapped the spine over some distance (marked by two small arrows) beyond the edge of the PSD (large arrows). Due to this progressive axonal wrapping of spine upon delayed perfusion fixation, images of spines being completely enwrapped (**C, D**) were often seen in single sections. Scale bar = 100 nm

Interestingly, upon delayed perfusion fixation, many more presynaptic terminals appear to extend beyond the active zone and enwrap around the cerebellar Purkinje spines (Fig. 5), with increasing length of the Type 3 PAP contact area (distance between two small arrows in Fig. 5A, B). Occasionally, this axonal wrapping appeared to be complete around the spine head in single sections (Fig. 5C, D), and such images were never seen in samples collected from fast perfusion fixation (cf. Figure 2A) where synapses were under resting states. Notably, similar type of axonal wrapping of spines was only rarely seen in cerebral cortex or in hippocampal CA1 stratum radiatum. Upon delayed perfusion fixation, occurrence frequency of presynaptic terminals completely ensheathing the postsynaptic spines in single sections pooled from 3 animals was 11.6% (13 out of 112 spines) in cerebellar molecular layer, and 1.1% (2 out of 180 spines) in hippocampal CA1 stratum radiatum.

## Disussion

The present study demonstrated stimulation-induced ultrastructural changes in peri-synaptic astrocytic processes (PAP) ensheathing the cerebellar Purkinje spines. Upon fast perfusion fixation, where synapses were under resting conditions, the majority of PAP’s were of Type 1 where the tip of the process was situated at the edge of the synaptic cleft. This perfectly appointed configuration of the tripartite synapse illustrates the advantageous location of the astrocytic process, poised to take up spilled out glutamate from the synaptic cleft. Upon a delay of 5–8 min in perfusion fixation, where synapses were under excitatory states, the occurrence frequency of this Type 1 PAP’s significantly decreased by more than half, accompanied by increases in both Type 2 and Type 3 PAP’s.

In Type 2 PAP, the astroglia covered part of the postsynaptic membrane that contains the PSD, where it is now separated from its presynaptic partner at the edge of the synaptic cleft. This separation of the pre-and post-synaptic elements was termed as “open cleft”, shown to be activity induced [23], and interpreted as to facilitate clearing of neurotransmitters from the cleft to help prevent overstimulation. Open clefts in hippocampus and cerebral cortex displayed exposed edges of the synaptic cleft without close apposition of astrocytic process, making the open cleft patent to extracellular space. However, here in cerebellar molecular layer, the separated presynaptic terminal and the PSD-containing postsynaptic membrane were always closely apposed by astrocytic processes which could quickly take up glutamate spilled out from the synaptic left. This configuration would also physically prevent the transmitter molecules from reaching the concentrated receptors at the PSD of the open cleft, reducing the probability of overstimulation.

The Type 3 PAP are located some distance away from the synaptic cleft, with the presynaptic axon terminals extending beyond the synaptic cleft and closely enwrapping the Purkinje spine. Upon delayed perfusion fixation, although the occurrence frequency of Type 3 PAP only increased slightly, the distance covered by the axon terminal over the spine beyond the synaptic cleft significantly increases to more than double. In some instances, the Purkinje spine heads appeared to be completely enwrapped by the presynaptic terminal in single sections. Although this phenomenon was not unique to cerebellar Purkinje spines, but it certainly occurred much more frequently than in hippocampus. Earlier studies have shown presence of glutamate transporters and glutamate uptake in hippocampal excitatory axon terminals [27–29]. Thus, it is possible that the extended axon terminals contain glutamate transporters on their plasma membrane to take up glutamate that spill out of the synaptic cleft during stimulation. If so, this configuration could also help prevent overstimulation. This speculation is consistent with precedents of other neurotransmitter transporters located on axolemma, such as GABA transporter [30], dopamine transporter [31] and serotonin transporter [32]. This kind of arrangement can also conserve and reuse neurotransmitters by local axonal reuptake. Notably, all three types of PAP can co-exist in a single synaptic junction and could work together in regulate glutamate uptake. A future 3D reconstruction of serial EM images may yield more detailed analysis on their exact configuration.

Interestingly, a subtype of glutamate transporter, EAAT4, is present on plasma membrane of Purkinje spines, but low at hippocampal pyramidal cell spines [5]. Thus, Purkinje spines appear to be equipped with one more way to take up glutamate. Furthermore, the Purkinje spine is unique in that its spine head is completely ensheathed by astroglia and its own presynaptic terminal under resting or stimulated conditions, although the proportion of coverage by these two elements may change depending on the activity states. Upon stimulation, (1) PAP’s extends into open cleft in Type 2, and (2) the presynaptic axon terminal extends beyond the synaptic cleft to wrap the Purkinje spine in Type 3. It is not clear why these seemingly contradictory mechanisms coexist that would trigger the extension of either astrocytic or axonal process, and the concurrent retraction of the other element. It is possible that Type 2 may be an early response while Type 3 has a later onset. Regardless, based on structural configuration alone, these complete ensheathing arrangements would limit the patency of the synaptic cleft to the extracellular space, and thus, seemingly impeded the transmitters diffusing out of the cleft. However, the abundance of glutamate transporters on plasma membranes of astroglia (EAAT1 & 2) and Purkinje spine (EAAT4) [5], and the possible presence of glutamate transporters on axon terminal plasma membrane (EAAT2) [29] could all quickly take up spilled out glutamate. This strategy of clearing transmitters totally by glutamate uptake is different from that for synapses in hippocampus or cerebral cortex where the clearing of transmitters is also facilitated by glutamate diffusion out of the open cleft. Thus, tripartite synapses in different regions of the brain have different strategies to clear out transmitters after acute stimulation.

## Electronic supplementary material

Below is the link to the electronic supplementary material.

Supplementary Material 1

## Data Availability

No datasets were generated or analysed during the current study.
