# Supplementary material for: Ultrastructural characterization of peri-synaptic astrocytic processes around cerebellar Purkinje spines under resting and stimulated conditions
Source: Mol Brain. 2025 Mar 31;18:28. doi: 10.1186/s13041-025-01198-7 (PMC11956224; doi:10.1186/s13041-025-01198-7)
Supplement: Supplementary file 1 — Supplementary Material 1 [file 13041_2025_1198_MOESM1_ESM.pdf]

Supplementary Fig. 1

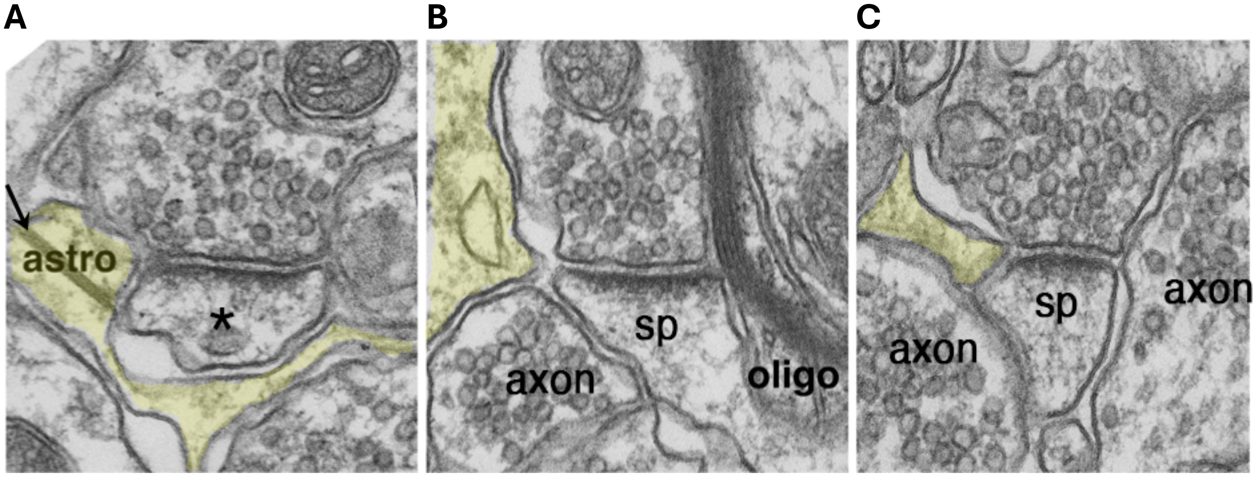

Examples of peri-synaptic astroglial processes (shaded in yellow) in hippocampal CA1 stratum radiatum. (A) Astrocytic processes with a characteristic gap junction (arrow) completely enwrapped this spine (asterisk). (B, C) Some spines (sp) were only partially ensheathed by astrocytic processes, while parts of the spine membranes were next to oligodendrocyte (B) or other neuronal elements (e. g., axon terminals in B & C).
